# Misunderstanding the Match: Do Students Create Rank Lists Based on True Preferences?

**DOI:** 10.5811/westjem.2019.10.44308

**Published:** 2020-12-09

**Authors:** Benjamin H. Schnapp, Kathleen Ulrich, Jamie Hess, Aaron S. Kraut, David Tillman, Mary Westergaard

**Affiliations:** University of Wisconsin School of Medicine and Public Health, BerbeeWalsh Department of Emergency Medicine, Madison, Wisconsin

## Abstract

**Introduction:**

The “stable marriage” algorithm underlying the National Residency Match Program (NRMP) has been shown to create optimal outcomes when students submit true preference lists. Previous research has shown students may allow external information to affect their rank lists. The objective of this study was to determine whether medical students consistently make rank lists that reflect their true preferences.

**Methods:**

A voluntary online survey was sent to third-year students at a single midwestern medical school. Students were given hypothetical scenarios that either should or should not affect their true residency preferences and rated the importance of six factors to their final rank list. The survey was edited by a group of education scholars and revised based on feedback from a pilot with current postgraduate year 1 residents.

**Results:**

Of 175 students surveyed, 140 (80%) responded; 63% (88/140) reported that their “perceived competitiveness” would influence their rank list at least a “moderate amount. Of 135 students, 31 (23%) moved a program lower on their list if they learned they were ranked “low” by that program, while 6% (8/135) of respondents moved a program higher if they learned they were ranked “at the top of the list.” Participants responded similarly (κ = 0.71) when presented with scenarios asking what they would do vs what a classmate should do.

**Conclusion:**

Students’ hypothetical rank lists did not consistently match their true residency preferences. These results may stem from a misunderstanding of the Match algorithm. Medical schools should consider augmenting explicit education related to the NRMP Match algorithm to ensure optimal outcomes for students.

## INTRODUCTION

The National Residency Match Program (NMRP) algorithm has been shown to create optimal outcomes for students when students and programs submit true preference lists.^1^ Despite this, previous research and anecdotal reports suggest that students may allow external information, such as perceived competitiveness, to influence their rank lists. This may be because historically it was possible to “game” the Match, although as of the most recent revision in 1998, this is no longer true.^2^

Multiple studies have shown that students alter their rank lists based on post-interview communication with programs. In a study of over 800 students across multiple specialties, 23.4% of respondents reported changing their rank order list based on communications with programs.^3^ One cross-sectional study of emergency medicine applicants found that 51% changed their rank lists based on post-interview communication.^4^ More recently, an online thread on *Reddit* entitled “How to Game the Match. Rank List Tips!” relayed stories of students stating “how dangerous it was to rank ‘reach’ programs higher.”^5^

To date however, there has been no study specifically examining the factors that medical students weigh to create rank lists. Our hypothesis was that medical students would be influenced in their creation of a hypothetical rank list by external information that did not affect their true residency preferences, suggesting a fundamental misunderstanding of the core principles of the NRMP Match algorithm.

## METHODS

The study was conducted at the University of Wisconsin School of Medicine and Public Health in Madison, Wisconsin, with 663 total students. All actively enrolled students in their third year of medical school (175 students) were eligible to participate.

To test students’ understanding of the Match algorithm, we created two types of case scenarios. One set of scenarios presented information that should cause a student to alter their true residency preferences and therefore their rank list, such as a partner securing a dream job in a new city or an ill family member. The other set of scenarios presented information that should not alter students’ true residency preferences or their rank lists, such as learning that they were highly competitive or would be low on a residency rank list. These case scenarios were developed to represent real-life scenarios that students might encounter as closely as possible to enhance content validity. Each type of scenario was presented in two ways: a “personal” scenario where students were asked what they *would* do if they were presented with this situation, and a “peer” scenario where they were asked to weigh in on what another student *should* do.

These case scenarios, as well as several questions about factors important in developing a rank list to provide internal structure validity, were developed by a group of three experienced education researchers within the Department of Emergency Medicine at the University of Wisconsin School of Medicine and Public Health. The scenarios were then piloted with several postgraduate year (PGY) 1 residents who had recently completed the Match process, and minor revisions were made for clarity and understanding for response process validity. While no formal assessment was made of consequences validity, third-year students were chosen as understanding the NRMP Match process is highly consequential to them during this period, while they remain unbiased by personal and peer experience with the Match. We did not explore relationships with other variables validity evidence in this study.

The combined instrument (Appendix A) was then emailed to the class email list as a voluntary, uncompensated Qualtrics (Provo, UT) survey in November 2018. Two reminder emails were sent approximately two weeks apart after the initial solicitation. The survey response rate used the second definition of response rate provided by the American Association for Public Opinion Research.^6^ We also conducted a wave analysis using Microsoft Excel (Redmond, WA) to determine whether nonresponse bias was present by comparing intial respondents with late respondents.^7^ An unweighted kappa was calculated using SPSS (Armonk, NY) between participants’ responses to “personal” and “peer” scenarios as a proxy for test-retest reliability and further evidence of internal stucture validity, since responses should not change based on the framing of the scenario. The study was determined to be exempt by the University of Wisconsin School of Medicine and Public Health Institutional Review Board.

## RESULTS

A total of 140/175 (80%) potential respondents completed at least the first section of the survey, and 131/175 (75%) respondents completed the survey in its entirety. Of these, 63% (88/140) reported that their “perceived competitiveness” would influence their rank list at least a “moderate amount.”

When presented with scenarios that should influence a rank list, 90% (122/135) of respondents would move a program higher on their list if they learned their significant other could only work in that program’s city, while 83% (112/135) of respondents would move a program lower on their list if they learned that the program director, who was their the sole reason for their interest in that program, was retiring. When asked to advise a friend on scenarios that should influence a rank list, 96% (126/131) advised that they should move a program up their rank list to be closer to an ill parent and 77% (101/131) advised that they should move a program down their rank list if a global health director, who was the sole reason for their interest in the program, was leaving.

When presented with hypothetical scenarios that should not influence a rank list, 23% (31/135) of respondents would move a program lower on their list if they learned they were ranked “low” by that program, while 6% (8/135) of respondents would move a program higher on their list if they learned they were ranked “at the top of the list” by that program. When asked to advise a friend on scenarios that should not influence a rank list, 9% (12/131) advised that that they should move a program up their rank list in response to a phone call from a coordinator indicating that they were a top applicant and ranked to match, and 22% (29/131) advised that they should move a program down their rank list when told that they would be low on the rank list at that program. The wave analysis on the “perceived competitiveness” question showed a minimal difference between responders and late responders (−0.02 on a five-item Likert scale) indicating a low likelihood of nonresponse bias. The unweighted kappa between analogous “personal” and “peer” scenarios across all raters was 0.71, indicating good agreement.

## DISCUSSION

Overall, our data suggest that there may be imperfect alignment of the Match algorithm design with student behavior. Many students failed to adjust rank lists appropriately according to new information that should have changed their true residency preferences, and a significant number also adjusted rank inappropriately based on “competitiveness” information that should not have affected their lists. These behaviors are inconsistent with the functioning of the NRMP’s matching algorithm^8^ and may put students and programs at risk for suboptimal Match outcomes.

One possible explanation is that students simply do not understand how the Match algorithm operates. The NRMP’s own video on the subject takes nearly five minutes to fully explain its workings,^9^ and the original paper detailing the algorithm runs on for seven highly technical pages.^1^ A lack of solid grounding in how the algorithm functions may lead to students leaning instead on hearsay and inherited wisdom. Further complicating the issue is that the Match has not always worked the same way, previously prioritizing the preferences of programs over those of applicants.^10^ While many resources for medical students offer good advice on how to construct a rank list correctly, this advice may be drowned out by the volume of suggestions that students are offered during this time period about seeking out mentorship, post-interview communications, and what factors are most important in choosing a residency.^11^,^12^ Medical schools and student advisors may need to make efforts to explicitly address the Match and how to create a proper rank list in order to avoid giving students an unappreciated disadvantage at this important training crossroads.

Of particular note, new information regarding competitiveness (both positive and negative) influenced students’ rank lists, when it should not have if students were attempting to obtain optimal outcomes from the Match algorithm. It is possible that the *knowledge* of being high or low on the rank list changes true preferences in some way, such as enhancing or detracting from a subjective assessment of fit. It has previously been shown that being liked improves one’s perception of the liker.^13^ However, the extent of this phenomenon in the Match process is unknown. It is also interesting that the percent of students deciding to alter their rank list is not the same when applicants are told that a program is ranking them highly compared to when a program is ranking them low (9% vs 23%). This may be related to the specifics of each scenario or represent an attempt at loss-aversion.^14^ These results also may suggest the importance of post-interview contacts from programs to applicants, as it appears that competitiveness information may influence student decision-making.

A commitment to the Match algorithm in the purest sense would require students and programs to keep competitiveness and rank information strictly confidential. However, the high-stakes pressures on both students and programs to find outstanding mutual compatibility likely will make this a difficult goal to achieve.

## LIMITATIONS

This study has several limitations. Of primary concern is the lack of a gold standard for rank-list behavior according to our specific scenarios. Further, our questions may not have been interpreted by the students as we intended. However, as the reliability was good between analogous scenarios, and the results from the scenarios are consistent with the finding from the first portion of the survey that a large percentage of students would be willing to change their rank list based on perceived competitiveness, we believe that it is likely the questions were understood as posed. Additionally, our study is a cross-sectional survey of a single class within a single medical school and may not be representative of medical students at other institutions or in other parts of the country.

## CONCLUSION

Nearly a quarter of students alter hypothetical rank lists based on information that should not affect their true residency preferences. As responses did not differ when asking students what they would do versus what a classmate should do, it is likely these results stem from a lack of understanding of the Match algorithm. Medical schools should consider adding explicit teaching related to the NRMP Match to ensure optimal outcomes for students.

## Supplementary Information


