# Supplementary material for: Misunderstanding the Match: Do Students Create Rank Lists Based on True Preferences?
Source: West J Emerg Med. 2020 Dec 9;21(1):4–7. doi: 10.5811/westjem.2019.10.44308 (PMC6948682; doi:10.5811/westjem.2019.10.44308)
Supplement: Supplementary file 1 [file wjem-21-4-s001.docx]

**APPENDIX A** (List of Questions Presented to Students)

After you complete your residency interviews this year, you will put together your final rank list, indicating your preference for matching at each residency.

We are interested in determining what influences medical students when they create their rank lists.

How much importance will each of the following factors have when placing residencies on your final rank list?

Reputation of the residency

- A great deal
- A lot
- A moderate amount
- A little
- None at all

Residency location (geography)

- A great deal
- A lot
- A moderate amount
- A little
- None at all

Quality of the faculty (including program director)

- A great deal
- A lot
- A moderate amount
- A little
- None at all

Quality of the residents (current residents and alumni)

- A great deal
- A lot
- A moderate amount
- A little
- None at all

Perceived competitiveness for the program (how likely you are to match)

- A great deal
- A lot
- A moderate amount
- A little
- None at all

Residency curriculum (educational experiences, rotations, didactics, simulation)

- A great deal
- A lot
- A moderate amount
- A little
- None at all

You receive a text message from your significant other.  They have just secured their dream job in City A.  You would like to be located as close to them as possible during residency.  Residency Program X is the only residency in City A and is currently third on your rank list.

You **plan to**:

- Move Residency Program X higher on your rank list
- Make no changes to your rank list
- Move Residency Program X lower on your rank list

You hear from a resident at Residency Program X that the program had an extremely competitive group of applicants this year and you will be at the bottom of their rank list.  Residency Program X is currently third on your rank list.

You **plan to**:

- Move Residency Program X higher on your rank list
- Make no changes to your rank list
- Move Residency Program X lower on your rank list

You hear reliable information from a colleague at Residency Program X that their longtime program director is retiring.  You were extremely fond of this program director and had placed Residency Program X third on your rank list based solely on the presence of this program director at Residency Program X.

You **plan to**:

- Move Residency Program X higher on your rank list
- Make no changes to your rank list
- Move Residency Program X lower on your rank list

You receive an email from your trusted research advisor.  She states that her reliable contact on faculty at Residency Program X mentioned that you will be at the very top of their rank list.  Residency Program X currently sits third on your rank list.

You **plan to**:

- Move Residency Program X higher on your rank list
- Make no changes to your rank list
- Move Residency Program X lower on your rank list

Bradley Badger hears reliable information from a colleague at Residency Program X that the program is prioritizing applicants from their own medical school this year and therefore will be ranking him towards the bottom of their rank list.  Residency Program X is currently third on Bradley’s rank list.

In order to have the best chance of matching at one of his most preferred programs, Bradley **should**:

- Move Residency Program X higher on his rank list
- Make no changes to his rank list
- Move Residency Program X lower on his rank list

Bucky Badger receives a phone call from his family.  One of his parents has become seriously ill, and he would now like to spend residency closer to them.  Residency Program X, currently third on his rank list, is the closest program to his parents.

In order to have the best chance of matching at one of his most preferred programs, Bucky **should**:

- Move Residency Program X higher on his rank list
- Make no changes to his rank list
- Move Residency Program X lower on his rank list

Bella Badger hears reliable information from a colleague at Residency Program X that their longtime Director of Global Health is leaving the institution.  Bella is extremely interested in pursuing Global Health and placed Residency Program X third on her rank list based solely on the presence of this faculty member at Residency Program X.

In order to have the best chance of matching at one of her most preferred programs, Bella **should**:

- Move Residency Program X higher on her rank list
- Make no changes to her rank list
- Move Residency Program X lower on her rank list

Betty Badger receives a phone call from Residency Program X, currently third on her rank list.  The program coordinator notes that she is one of their top applicants this year and that she is ranked to match.

In order to have the best chance of matching at one of her most preferred programs, Betty **should**:

- Move Residency Program X higher on her rank list
- Make no changes to her rank list
- Move Residency Program X lower on her rank list
